# Evaluating the Impact of Mixed-Reality Technology on Operating Room Time in Total Hip Arthroplasty: A Comparative Study

**DOI:** 10.1016/j.artd.2025.101734

**Published:** 2025-06-10

**Authors:** Crystal Jing, Matthew K. Stein, David G. Deckey, Michael P. Bolognesi, Samuel S. Wellman, Sean P. Ryan

**Affiliations:** a School of Medicine, Duke University, Durham, NC, USA; b Department of Orthopaedic Surgery, Duke University Medical Center, Durham, NC, USA

**Keywords:** Total hip arthroplasty, Surgical navigation technology, Operative time, Mixed-reality, Augmented reality

## Abstract

**Background:**

To help guide acetabular component positioning in total hip arthroplasty (THA), computer-assisted devices like robotics, navigation, and mixed-reality (MR) have been incorporated into the operating room with variable results. This study aimed to identify the effect of using MR technology on operative time.

**Methods:**

This was a retrospective review of patients over the age of 18 years, who underwent primary THA for end-stage osteoarthritis from January 1, 2021, to August 20, 2024, at a single institution with 2 surgeons who incorporated MR into their surgical workflow. Patients that underwent THA with one of these 2 surgeons without HipInsight (Surgical Planning Associates, Inc., Boston, MA) were also included as controls. Demographic data, surgical approach, and operative time were evaluated.

**Results:**

There were 411 patients included in this study with 165 patients who underwent surgery with MR and 246 patients who underwent surgery with standard manual instrumentation. The mean operative time in minutes was 89.6 (standard deviation = 12.6) for patients undergoing THA without MR and 89.2 (standard deviation = 13.1) for patients undergoing THA with MR (*P* > .05).

**Conclusions:**

A MR navigation system for THA did not appear to prolong operative times when utilized. Further studies are needed to determine its effect on long-term outcomes.

## Introduction

Total hip arthroplasty (THA) is most often indicated for patients with end-stage osteoarthritis (OA). One of the challenges faced by surgeons during THA is correct positioning of the acetabular component with optimal anteversion and inclination angles [1,2]. Malpositioning may lead to complications, such as dislocation and accelerated bearing wear [3,4]. Historically, Lewinnek et al. [5] has been cited for their description of the “safe zone” with inclination of 40° ± 10° and anteversion 15° ± 10° for patients without abnormal spinopelvic relationships. Alternatively, the Callanan safe zone is defined as 30°-45° of inclination and 5°-25° of anteversion [6], while Dorr and Callaghan published an article critiquing the Lewinnek safe zone, commenting on its poor accuracy [7]. These studies highlight the importance of the hip-spine relationship and sagittal plane [8], which may lead to different intraoperative targets for idealized component positioning in different patients. Nonetheless, the accuracy of intraoperative component positioning and ability to hit inclination and anteversion targets have proven difficult.

Historically, the most implemented strategy for placement of the acetabular component has been the use of a mechanical guide. Unfortunately, prior authors have shown there to be significant variation in cup alignment from the optimal abduction and version angles with the use of mechanical guides [9]. Another method is freehand positioning, which many have argued to be inferior to the use of mechanical guides. Bosker et al. [3] reported that the freehand method often led to underestimation of abduction and anteversion. Many studies have demonstrated the inconsistency in acetabular cup positioning via freehand and mechanically guided methods and show promise for the integration of computer-assisted technologies in THA [10].

There exist a variety of technologies to aid in the efficiency and accuracy of acetabular cup placement in THA, including both computer-assisted and robot-assisted systems. Preoperative computed tomography scans are often obtained and used as an intraoperative guide. Fluoroscopy-guided technologies have also been implemented, especially in anterior-based approach THA [11,12]. Jacob et al. [13] showed that computer-assisted THA systems demonstrate superior accuracy in cup positioning as compared to freehand placement. Likewise, Agarwal et al. [14] reported that the use of computer-assisted navigation systems was associated with lower postoperative dislocation rates. However, the most frequent critique of these navigation systems is the reported increase in intraoperative time [[15], [16], [17]]. A specific example of assistive technology is OrthoGrid Hip AI (Zimmer Biomet, Warsaw, IN), which is designed for direct-anterior-approach THA and provides intraoperative feedback and tools to assist in pelvic plane tracking, offset, and leg length and cup positioning [16].

More recently, mixed-reality (MR) systems have become incorporated into the operating room [18]. The HipInsight System (Surgical Planning Associates, Inc., Boston, MA) is one such MR-guided platform for THA. This MR system utilizes a HoloLens 2 (Microsoft Corporation, Redmond, WA) to provide surgeons with three-dimensional holograms of a patient’s anatomy, implants, and instruments to allow accurate and efficient visualization of acetabular component alignment. One of the proposed disadvantages of computer-based systems during THA is increasing operative time.

At the investigating institution, MR was incorporated into 2 arthroplasty surgeons’ practices. This study aimed to evaluate the impact of 1 MR system on operating room time for THA.

## Material and methods

### Study population

**Figure 1:**
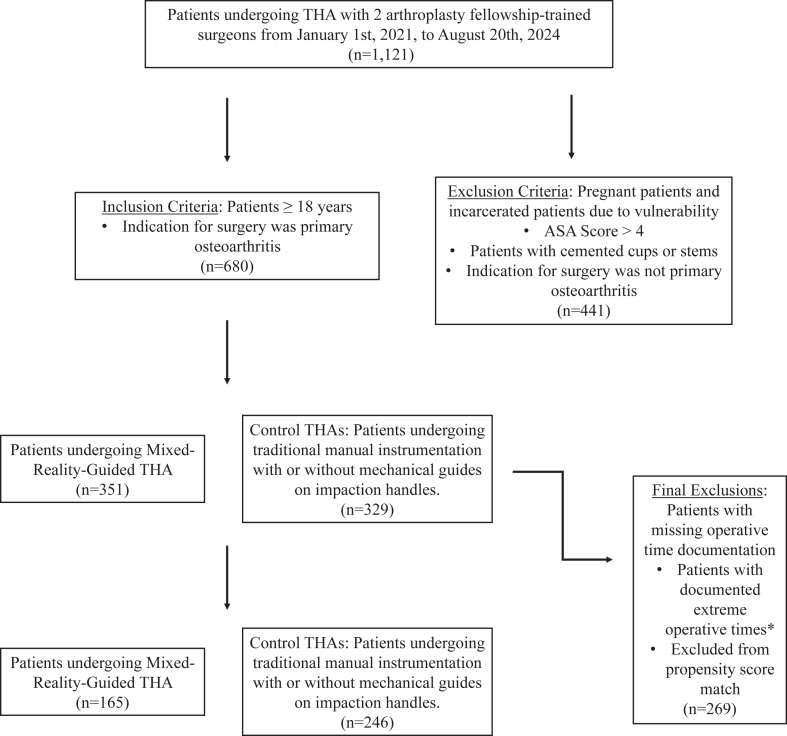
Inclusion and exclusion criteria for patients in this study. ASA, American Society of Anesthesiologists; THA, total hip arthroplasty.

This study was determined exempt by the institutional review board. Patients were identified using an institutional database. Patients were included if they were at least 18 years of age and have undergone primary THA between January 1, 2021, and August 20, 2024, for the indication of primary OA with one of 2 arthroplasty fellowship-trained surgeons who incorporated MR with HipInsight technology (Surgical Planning Associates, Inc.) into their practices. Inclusion and exclusion criteria are depicted in Figure 1.

In the absence of MR, each surgeon utilized traditional manual instrumentation with or without mechanical guides on impaction handles. Operative times for surgeries with and without MR were compared.

There were 681 patients who underwent primary THA that met inclusion criteria, with 351 patients who underwent surgery with MR and 329 patients who underwent surgery without MR. Given the varying complexity of cases noted at a tertiary referral center, there was a wide range of operative times documented, both fast and slow, with some patients having no operative start time or complete time documented. For this reason, the outlying operative times present in the lowest and highest quartile were eliminated, allowing for evaluation of surgery time most noted for a standard primary THA for OA. Exclusion of the lowest and highest quartiles allowed for mitigation of distracting outliers on the mean in this non-normally distributed dataset.

Following this exclusion, there were 351 MR patients and 329 no MR control patients. Then, control and MR patients were propensity score matched with a 2:1 ratio using a 0.2 caliper with the “nearest neighbor” method. Covariates used were body mass index, American Society of Anesthesiologists score, age, and sex. This yielded a final cohort of 411 patients with 165 undergoing THA with MR and 246 undergoing THA without MR (Fig. 1).

### Demographics

Demographic data, including age, gender, race, and ethnicity, were recorded. The mean age of patients who underwent THA without MR was 65.5 (standard deviation [SD] = 11.2) years, and 65.3 (SD = 10.4) years for those who underwent THA with technology (*P* > .05). Sex, race, ethnicity, American Society of Anesthesiologists score, and body mass index were similar between groups (Table 1).

**Table 1:** Demographics and procedure details.

Demographics	No mixed-reality usage (n = 246)	Mixed-reality usage (n = 165)	*P*-value
Age (mean [SD])	65.5 (11.2)	65.3 (10.4)	.616
Gender (n [%])			
Men	108 (43.9)	77 (46.7)	.614
Women	138 (56.1)	88 (53.3)	
Race (n [%])			.446
Asian & Pacific Islander	1 (0.4)	1 (0.6)	
Black	51 (20.7)	2 (1.2)	
White	189 (76.8)	36 (21.8)	
American Indian, Alaskan Native, Native Hawaiian	0 (0.0)	1 (0.6)	
Other	2 (0.8)	4 (2.4)	
NR	3 (1.2)	1 (0.6)	
Ethnicity (n [%])			.484
Non-Hispanic	240 (97.6)	163 (98.8)	
Hispanic	6 (2.4)	2 (1.2)	
American Society of Anesthesiologists (ASA) Score			.639
I	2 (0.8)	2 (1.2)	
II	127 (51.6)	91 (55.2)	
III	117 (47.6)	72 (43.3)	
IV	0 (0.0)	0 (0.0)	
Body mass index, kg/m^2^ (mean [SD])	30.42 (5.6)	30.68 (6.9)	.848
Procedure details			
Operative time (mean [SD])	89.6 (12.6)	89.2 (13.1)	.823
Operative time (median [IQR])	88 (81, 97)	89 (79, 96)	–

IQR, interquartile range; NR, not reported; SD, standard deviation.

### Operative planning and technique

All patients evaluated for THA were offered the option of MR-guided procedure. However, patients who did not obtain a preoperative computed tomography scan required for planning of MR or personal preference underwent standard techniques.

The use of MR requires only the assembly of a single reusable array on the back table. Operative plan was available to review on a portable computer before the case if desired. A standard posterior approach was undertaken for all cases in this study. Sequential reaming to 1 mm under the final diameter of planned acetabular component was undertaken until there was evidence of bleeding bone circumferentially. The final cup size was then provisionally impacted in an anatomic position. A targeting array with a Quick Response code is secured to the patient’s anterior superior iliac spine, ilium, and ischium per instrument protocol [[19], [20], [21], [22]]. As previously described by Dilbone et al. [20] and instructed by the technique guide for the system, a long pin is drilled into the ischium using an ischial guide provided by the MR navigation system. Next, the anterior superior iliac spine was identified for positioning of the antero-superior trocar, and the ilium was identified for placement of the postero-superior trocar. The application of the array, long pin, and 2 trocars takes less than 3 minutes [20]. The HoloLens 2 MR (Microsoft Corporation) eyewear was then donned on the attending surgeon in a sterile fashion. MR-guided technology of eyewear was then used to visualize acetabular cup placement in reference to the planned inclination and anteversion (typically 43° and 20°, respectively; the acetabular cup was then adjusted to align with the preoperatively planned orientation if discrepancy existed). Quick Response array and MR goggles were then removed. Screws and trial or final liner were placed based on surgeon preference. The femur was then prepared. After the trial femoral component was in place, an intraoperative radiograph was obtained to check appropriate component size and position (regardless of whether MR was used). The trial components were then removed, and the final stem and head components were placed. Standard capsular closure and skin closure were then performed.

### Operative measures

The primary outcome of this study was operative time, which was defined as the time from the start of the procedure to the time that the procedure was completed as noted in the operative reports in our institution’s electronic medical record as charted by circulating nurses during the procedures. Procedure start was defined as the time of incision. Procedure finish time was defined as the time skin closure was completed and drapes were taken down. Other data that were collected in our study included the operative approach and the use of cement; this information was obtained from manual chart review of operative reports.

### Data analyses

Descriptive statistics were conducted using RStudio version 4.2.2 (Posit Software, Boston, MA). Continuous variables were described using either means and SD or medians with interquartile ranges. Categorical variables were described using proportions. Continuous variables were compared between groups via *t*-test for parametric data and Wilcoxon Rank Sum test for nonparametric data. Categorical variables were compared via chi-square tests. Multiple linear regression analysis was used to determine the role of MR usage and demographic predictors on operative time. Bonferroni correction was not required in this regression analysis as only a single outcome was studied. A *P*-value of < .05 was set to indicate statistical significance. Generalized additive model regression was utilized for visualizing operative time trends over the years of study data collection.

## Results

### Operative time

The mean operative time in minutes was 89.2 (SD = 13.1) for patients undergoing THA with MR and 89.7 (SD = 12.7) for patients undergoing THA without MR (*P* > .05). The median operative time in minutes was 89 [interquartile range 79, 96] for MR patients and 88 [interquartile range 81, 87] for traditionally instrumented patients (Table 1).

Eight different types of acetabular cups were used among patients, with a majority of patients receiving a Pinnacle acetabular cup (DePuy Synthes, Warsaw, IN) in both groups (*P* < .0001, Table 2, Table 3).

**Table 2:** Acetabular and femoral component implant data.

Implant manufacturer	No mixed-reality usage (n = 246)	Mixed-reality usage (n = 165)	*P*-value
Acetabular cup			**<.0001**
Pinnacle	166 (67.5)	76 (46.1)	
G7	42 (17.1)	44 (26.7)	
Platform	22 (8.9)	43 (26.1)	
EMPOWR	12 (4.9)	0 (0.0)	
EMPHASYS	0 (0.0)	2 (1.2)	
R3	1 (0.4)	0 (0.0)	
Trident II	1 (0.4)	0 (0.0)	
Klassic-HD	2 (0.8)	0 (0.0)	
Femoral stem			**.0034**
Summit	183 (74.4)	121 (73.3)	
Avenir	25 (10.2)	20 (12.1)	
Echo	15 (6.1)	20 (12.1)	
TaperFill	13 (5.3)	0 (0.0)	
Klassic	4 (1.6)	0 (0.0)	
Wagner Cone	5 (2.0)	3 (1.8)	
Taperloc	1 (0.4)	1 (0.6)	

Significance is denoted in bold if *P* < .05.

**Table 3:** Summary of implants used among patients.

Implant system	Manufacturer	Type	Material
Acetabular cup			
Pinnacle	DePuy Synthes	Conventional fixed-bearing	Titanium alloy
G7	Zimmer Biomet	Conventional fixed-bearing	Titanium alloy
Platform	Total Joint Orthopedics	Conventional fixed-bearing	Titanium alloy
EMPOWR	Enovis	Dual-mobility	Cobalt-chrome, polyethylene
EMPHASYS	DePuy Synthes	Conventional fixed-bearing	Titanium alloy
R3	Smith & Nephew	Conventional fixed-bearing	Oxidized zirconium alloy
Trident II	Stryker	Conventional fixed-bearing	Titanium (porous)
Klassic-HD	Total Joint Orthopedics	Conventional fixed-bearing	Titanium alloy
Femoral stem			
Summit	DePuy Synthes	Tapered, cementless	Titanium alloy
Avenir	Zimmer Biomet	Tapered, cementless	Titanium alloy
Echo	Zimmer	Tapered, cementless	Titanium alloy
TaperFill	Enovis	Tapered wedge, cementless	Titanium alloy
Klassic	Total Joint Orthopedics	Tapered, cementless	Titanium (porous)
Wagner Cone	Zimmer Biomet	Tapered, cementless	Titanium alloy
Taperloc	Zimmer Biomet	Tapered, cementless	Titanium (porous)

### Learning curve

**Figure 2:**
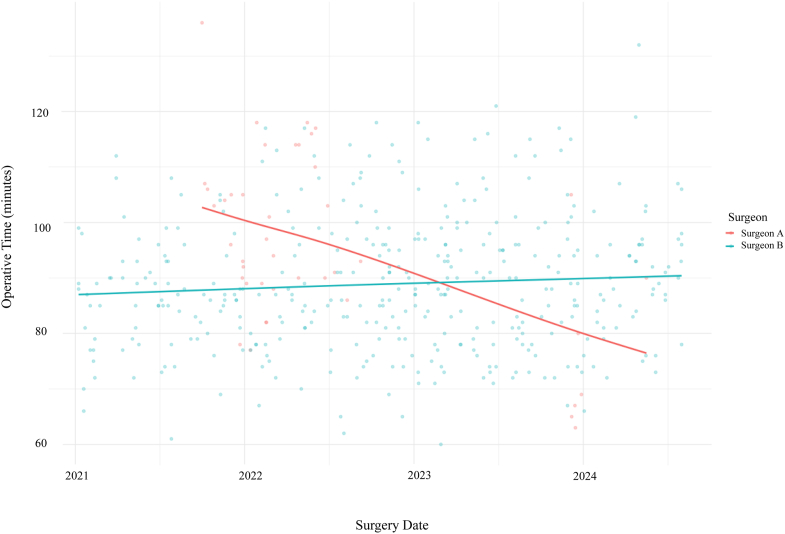
Learning curve using mixed-reality technology.

Both surgeons included in this study received training on this MR technology prior to implementation into their practice. Surgeon B utilized similar technologies for multiple years prior to the use of this specific MR technology. Surgeon A trained with Surgeon B prior to implementation into their practice. Thus, a steeper learning curve in Surgeon A’s operative times is shown in earlier years; however, after 1 year of MR usage, Surgeon A’s operative times were less than that of Surgeon B. Learning curves are demonstrated in Figure 2. In terms of overall surgeon experience level, upon study inception, Surgeon A was in their first year of practice, and Surgeon B was in their thirteenth year of practice.

## Discussion

In this study, the use of MR did not prolong operative time. MR utilization may help to optimize efficiency through reduction of trial and error with component positioning during freehand instrumentation, thereby reducing operative time; however, the statistically significant reduction in operating time is only seen in one of 2 surgeons. At minimum, the present study does not demonstrate prolonged surgical time with incorporation of this new technology, potentially offering the benefit of improved component accuracy without additional time costs, which have been observed with other computer-assisted systems.

Existing literature on navigation systems generally reports that some of these technologies prolong operative time, with some studies showing statistical significance, and others not. Kunze et al. [15] conducted a meta-analysis of randomized controlled trials to compare manual, robot-assisted, and computer-navigated THA. They found that manual THA yielded significantly shorter operative times as robot-assisted and computer-assisted THAs [15]. Similarly, Christ et al. [23] conducted a retrospective review of 61 patients undergoing primary THA by a single surgeon using an imageless navigation device and identified a mean 2.9-minute (SD = 1.6) increase in operative time per procedure as compared to manual THA; they proposed that setup and hands-on utilization of a navigation tool contributed to this slightly prolonged operative time. However, this study did not note any statistical significance in this observed difference in time (*P* = .60). In a meta-analysis of randomized control trials by Xu et al. [24], they found that the operative time was significantly longer for navigation system-assisted procedures than for manual THA. Literature specific to MR-navigation systems and operative time in THA is limited, and existing data are on total knee arthroplasty or vertebroplasty; these studies have shown that MR navigation does not prolong operative time, similar to the findings of the present study [25,26].

Despite varying significance among studies, the current literature consistently touts the advantages of these technologies in accuracy and precision of component placement in THA.

Prior studies of this MR technology have demonstrated other advantages. Ryan et al. [21] conducted a survey study and reported that the use of an MR system improved trainee’s understanding of component placement. A case report by Leal et al. [27] described the use of HipInsight (Surgical Planning Associates, Inc.) in guiding a successful conversion of surgical hip fusion to THA, noting the benefits of the MR technology in accounting for pelvic tilt, rotation, offset, leg-length discrepancy, obliquity, and patient anatomy in complex cases. A prior study from our institution utilizing the same MR technology found that the use of MR navigation led to 73.4% of acetabular cups adjusted at least 5° in any direction from placement without MR to then confirming the location of the cup with MR. In addition, they found that operative inclination was corrected at least 5° in 43% of cases following MR navigation when compared to placement of the cup using solely anatomic landmarks [19]. Despite these limited reports regarding education and complex cases, surgical time has not previously been evaluated for routine primary THA cases.

There are several important limitations to this study worth noting. The MR system was used in a single tertiary referral system by fellowship-trained adult reconstruction surgeons, and the results may not be generalizable to the general population. In addition, the authors recognize there are numerous variables that contribute to surgical efficiency including case complexity, surgeons’ experience with improved efficiency over time, and surgical staff’s experience and assistance. All these factors contribute to variations in operating room time and cannot be completely controlled for. The authors attempted to control for this by utilizing a large number of cases specifically for OA and eliminating outlying operative times. At minimum, this analysis contributes to the literature by demonstrating a lack of prolonged operative time with the incorporation of new technology.

This study is important for arthroplasty surgeons who are considering implementation of MR-based navigation tools in intraoperative settings. The benefits of this technology in THA preoperative planning and intraoperative visualization of anatomy and guidance with acetabular cup placement are promising. The possibility of increased efficiency using this technology may provide additional benefits of reduction in infection rates and possibly cost, although the few minutes in reduced operating room time noted in the current study are unlikely to significantly contribute to these variables.

## Conclusions

A MR navigation system for THA did not appear to prolong operative times when utilized, and greater confidence in positioning may contribute to a reduction in operative time at a single tertiary center. Further studies are needed to determine its effect on long-term patient outcomes.

## Conflicts of interest

MPB receives royalties from Smith & Nephew, Total Joint Orthopaedics, and Zimmer; is an unpaid consultant for Amedica; has stock or stock options with Amedica and Total Joint Orthopaedics; receives research support as a principal investigator (PI) from Biomet, Exactech, Inc, KCI, Zimmer, and DePuy; receives other financial or material support from Acelity and AOA Omega; is in the editorial or governing board of *Journal of Arthroplasty* and *Arthroplasty Today*; and is a board member of American Association for Hip and Knee Surgeons, Eastern Orthopaedic Association, and American Academy of Orthopaedic Surgeons. DGD has stock or stock options with Osgenic; is in the editorial or governing board of *Journal of Arthroplasty*; and is a board member of American Association for Hip and Knee Surgeons and American Academy of Orthopaedic Surgeons. SPR is a paid consultant for Zimmer and receives research support as a PI from Zimmer and Smith & Nephew. SSW is in the speakers bureau of/gave paid presentations for TJO and Zimmer; is a paid consultant for Smith & Nephew and TJO; has stock or stock options in Joint Development, LLC; receives research support as a PI from Biomet, DePuy, A Johnson & Johnson Company, Medacta, Smith & Nephew, Stryker, and Zimmer; receives royalties or financial or material support from TJO; is in the editorial/governing board of *Journal of Arthroplasty*; and is a board member of American Association of Hip and Knee Surgeons. All other authors declare no potential conflicts of interest.

For full disclosure statements, refer to https://doi.org/10.1016/j.artd.2025.101734.

## CRediT authorship contribution statement

**Crystal Jing:** Writing – review & editing, Writing – original draft, Visualization, Validation, Methodology, Investigation, Formal analysis, Data curation. **Matthew K. Stein:** Writing – review & editing, Writing – original draft, Visualization, Validation, Supervision, Software, Resources, Project administration, Methodology, Investigation, Conceptualization. **David G. Deckey:** Writing – review & editing, Visualization, Validation, Supervision, Software, Resources, Project administration, Methodology, Investigation, Conceptualization. **Michael P. Bolognesi:** Writing – review & editing, Visualization, Validation, Supervision, Software, Resources, Project administration, Methodology, Investigation, Conceptualization. **Samuel S. Wellman:** Writing – review & editing, Visualization, Validation, Supervision, Software, Resources, Project administration, Methodology, Investigation, Conceptualization. **Sean P. Ryan:** Writing – review & editing, Visualization, Validation, Supervision, Software, Resources, Project administration, Methodology, Investigation, Formal analysis, Data curation, Conceptualization.

## Appendix A Supplementary data

Conflict of Interest Statement for Bolognesi

Conflict of Interest Statement for Deckey

Conflict of Interest Statement for Jing

Conflict of Interest Statement for Ryan

Conflict of Interest Statement for Stein

Conflict of Interest Statement for Wellman

## References

[1] Paterno S.A., Lachiewicz P.F., Kelley S.S. (1997). The influence of patient-related factors and the position of the acetabular component on the rate of dislocation after total hip replacement. J Bone Joint Surg Am.

[2] Pierchon F., Pasquier G., Cotten A., Fontaine C., Clarisse J., Duquennoy A. (1994). Causes of dislocation of total hip arthroplasty. CT study of component alignment. J Bone Joint Surg Br.

[3] Bosker B.H., Verheyen C.C.P.M., Horstmann W.G., Tulp N.J.A. (2007). Poor accuracy of freehand cup positioning during total hip arthroplasty. Arch Orthop Trauma Surg.

[4] Patil S., Bergula A., Chen P.C., Colwell C.W., D’Lima D.D. (2003). Polyethylene wear and acetabular component orientation. J Bone Joint Surg Am.

[5] Lewinnek G.E., Lewis J.L., Tarr R., Compere C.L., Zimmerman J.R. (1978). Dislocations after total hip-replacement arthroplasties. J Bone Joint Surg Am.

[6] Callanan M.C., Jarrett B., Bragdon C.R., Zurakowski D., Rubash H.E., Freiberg A.A. (2011). The John Charnley Award: risk factors for cup malpositioning: quality improvement through a joint registry at a tertiary hospital. Clin Orthop Relat Res.

[7] Dorr L.D., Callaghan J.J. (2019). Death of the Lewinnek “safe zone.”. J Arthroplasty.

[8] DiGioia A.M., Jaramaz B., Colgan B.D. (1998). Computer assisted orthopaedic surgery. Image guided and robotic assistive technologies. Clin Orthop Relat Res.

[9] DiGioia A.M., Jaramaz B., Plakseychuk A.Y., Moody J.E., Nikou C., LaBarca R.S. (2002). Comparison of a mechanical acetabular alignment guide with computer placement of the socket. J Arthroplasty.

[10] Nishihara S., Hayashida K. (2022). Comparison between freehand technique and computed tomography-based navigation in acetabular cup placement through direct anterior approach for total hip arthroplasty. Arch Orthop Trauma Surg.

[11] Ong C.B., Buchan G.B.J., Acuña A.J., Hecht C.J., Homma Y., Shah R.P. (2023). Cost-effectiveness of a novel, fluoroscopy-based robotic-assisted total hip arthroplasty system: a Markov analysis. Int J Med Robot.

[12] Summers S., Ocksrider J., Lezak B., Zachwieja E.C., Schneiderbauer M.M. (2021). Intra-operative referencing technique is non-inferior to use of fluoroscopy for acetabular component positioning in anterior hip arthroplasty. J Clin Orthop Trauma.

[13] Jacob I., Benson J., Shanaghan K., Gonzalez Della Valle A. (2020). Acetabular positioning is more consistent with the use of a novel miniature computer-assisted device. Int Orthop.

[14] Agarwal S., Eckhard L., Walter W.L., Peng A., Hatton A., Donnelly B. (2021). The use of computer navigation in total hip arthroplasty is associated with a reduced rate of revision for dislocation: a study of 6,912 navigated THA procedures from the Australian orthopaedic association national joint replacement registry. J Bone Joint Surg Am.

[15] Kunze K.N., Bovonratwet P., Polce E.M., Paul K., Sculco P.K. (2022). Comparison of surgical time, short-term adverse events, and implant placement accuracy between manual, robotic-assisted, and computer-navigated total hip arthroplasty: a network meta-analysis of randomized controlled trials. JAAOS Glob Res Rev.

[16] Lim S.-J., Ko K.-R., Park C.-W., Moon Y.-W., Park Y.-S. (2015). Robot-assisted primary cementless total hip arthroplasty with a short femoral stem: a prospective randomized short-term outcome study. Computer Aided Surg.

[17] Foissey C., Batailler C., Coulomb R., Giebaly D.E., Coulin B., Lustig S. (2023). Image-based robotic-assisted total hip arthroplasty through direct anterior approach allows a better orientation of the acetabular cup and a better restitution of the centre of rotation than a conventional procedure. Int Orthop.

[18] Hasegawa M., Naito Y., Tone S., Sudo A. (2024). Comparison between accuracy of augmented reality computed tomography-based and portable augmented reality-based navigation systems for cup insertion in total hip arthroplasty. Sci Rep.

[19] Leal J., Heimann A.F., Dilbone E.S., Ryan S.P., Wellman S.S. (2025). How much does a computed tomography-based mixed-reality navigation system change freehand acetabular component position?. Arthroplast Today.

[20] Dilbone E.S., Heimann A.F., Leal J., Ryan S.P., Wellman S.S. (2025). Evaluating the accuracy of a computed tomography-based mixed-reality navigation tool for acetabular component positioning in total hip arthroplasty. J Arthroplasty.

[21] Ryan S., Cochrane N., Bolognesi M., Wellman S. (2024). Enhanced total hip arthroplasty education using augmented reality: a survey from a tertiary center. Orthopedics.

[22] Steppacher S.D., Kowal J.H., Murphy S.B. (2011). Improving cup positioning using a mechanical navigation instrument. Clin Orthop Relat Res.

[23] Christ A., Ponzio D., Pitta M., Carroll K., Muir J.M., Sculco P.K. (2018). Minimal increase in total hip arthroplasty surgical procedural time with the use of a novel surgical navigation tool. Open Orthop J.

[24] Xu K., Li Y., Zhang H., Wang C., Xu Y., Li Z. (2014). Computer navigation in total hip arthroplasty: a meta-analysis of randomized controlled trials. Int J Surg.

[25] Hu M.-H., Chiang C.-C., Wang M.-L., Wu N.-Y., Lee P.-Y. (2020). Clinical feasibility of the augmented reality computer-assisted spine surgery system for percutaneous vertebroplasty. Eur Spine J.

[26] Lambrechts J., Vansintjan P., Lapierre C., Sinnaeve F., Van Lysebettens W., Van Overschelde P. (2024). Accuracy of a new augmented reality assisted technique for total knee arthroplasty: an in vivo study. Arthroplast Today.

[27] Leal J., Cullen M.M., Bolognesi M.P., Wellman S.S., Ryan S.P. (2024). Mixed reality navigation in hip fusion conversion: a novel utilization of advanced technology: a case report. JBJS Case Connect.

