# Supplementary material for: Evaluating the Impact of Mixed-Reality Technology on Operating Room Time in Total Hip Arthroplasty: A Comparative Study
Source: Arthroplast Today. 2025 Jun 10;33:101734. doi: 10.1016/j.artd.2025.101734 (PMC12179719; doi:10.1016/j.artd.2025.101734)
Supplement: Conflict of Interest Statement for Bolognesi [file mmc1.docx]

# INDIVIDUAL CONFLICT OF INTEREST STATEMENT

***American Association of Hip and Knee Surgeons***

(Adopted from the American Academy of Orthopaedic Surgeons disclosure statement)

The following form **must be filled out completely and submitted by each author (example, 6 authors, 6 forms).**

**All items require a response. If there is no relevant disclosure for a given item, enter "*None*.”**

**TITLE**

1. Royalties from a company or supplier (The following conflicts were disclosed)

**Smith & Nephew; Total Joint Orthopaedics; Zimmer**

2. Speakers bureau/paid presentations for a company or supplier (The following conflicts were disclosed)

**None**

3A. Paid employee for a company or supplier (The following conflicts were disclosed)

**None**

3B. Paid consultant for a company or supplier (The following conflicts were disclosed)

**None**

3C. Unpaid consultants for a company or supplier (The following conflicts were disclosed)

**Amedica**

4. Stock or stock options in a company or supplier (The following conflicts were disclosed)

**Amedica; Total Joint Orthopaedics**

5. Research support from a company or supplier as a Principal Investigator (The following conflicts were disclosed)

**Biomet; Exactech, Inc; KCI; Zimmer; DePuy**

6. Other financial or material support from a company or supplier (The following conflicts were disclosed)

**Acelity; AOA Omega**

7. Royalties, financial or material support from publishers (The following conflicts were disclosed)

**None**

8. Medical/Orthopaedic publications editorial/governing board (The following conflicts were disclosed)

**Journal of Arthroplasty; Arthroplasty Today**

9. Board member/committee appointments for a society (The following conflicts were disclosed)

**AAHKS; EOA; AAOS**

**Each author must sign AND print or type his/her name, date and submit a separate form**

In addition, one BLINDED Conflict of Interest form (no author names used) should be submitted per manuscript with all author disclosures.

Michael P. Bolognesi MICHAEL P. BOLOGNESI 07/12/2024

Author Name (Print or Type) Author Signature Date
