# Supplementary material for: Evaluating the Impact of Mixed-Reality Technology on Operating Room Time in Total Hip Arthroplasty: A Comparative Study
Source: Arthroplast Today. 2025 Jun 10;33:101734. doi: 10.1016/j.artd.2025.101734 (PMC12179719; doi:10.1016/j.artd.2025.101734)
Supplement: Conflict of Interest Statement for Wellman [file mmc6.docx]

# INDIVIDUAL CONFLICT OF INTEREST STATEMENT

***American Association of Hip and Knee Surgeons***

(Adopted from the American Academy of Orthopaedic Surgeons disclosure statement)

The following form **must be filled out completely and submitted by each author (example, 6 authors, 6 forms).**

**All items require a response. If there is no relevant disclosure for a given item, enter "*None*.”**

**Management of Diabetic Oral Medications After Primary Total Knee Arthroplasty**

**Manuscript Title**

1. Royalties from a company or supplier: NONE

2. Speakers bureau/paid presentations for a company: TJO, Zimmer

3A. Paid employee for a company or supplier: NONE

3B. Paid consultant for a company: Smith & Nephew, TJO

3C. Unpaid consultants for a company or supplier: NONE

4. Stock or stock options in a company or supplier: Joint Development, LLC

5. Research support from a company or supplier as a Principal Investigator: Biomet, DePuy, A Johnson & Johnson Company, Medacta, Smith & Nephew, Stryker, Zimmer

6. Other financial or material support from a company or supplier: NONE

7. Royalties, financial or material support from publishers: TJO

8. Medical/Orthopaedic publications editorial/governing board: Journal of Arthroplasty

9. Board member/committee appointments for a society: American Association of Hip and Knee Surgeons

Samuel Wellman Samuel Wellman 1/4/2025

Author Name (Print or Type) Author Signature Date
